# Knowledge and attitude towards cervical cancer among reproductive age group women in Gondar town, North West Ethiopia

**DOI:** 10.1186/s12889-020-8229-4

**Published:** 2020-02-11

**Authors:** Ayelign Mengesha, Anteneh Messele, Biruk Beletew

**Affiliations:** 1 College of Health Sciences, Department of Nursing, Woldia University, Weldia, Ethiopia; 2 School of Nursing and Midwifery, Department of Nursing, University of Gondar, Gondar, Ethiopia; 3 College of Health Sciences, Department of Nursing, Woldia University, Weldia, Ethiopia

**Keywords:** Cervical Cancer, Knowledge, Attitude, Reproductive age group women, Gondar town

## Abstract

**Background:**

Cervical cancer is the second most commonly diagnosed cancer and the third leading cause of cancer death in women worldwide. Nearly 83% of the world’s new cases and 85% of all cervical cancer-related deaths occur in developing countries. It is primarily caused by human papilloma virus (HPV); a sexually transmitted pathogen that could be prevented with safe sexual practice and using vaccines among others.

The aim of the study was to assess the knowledge and attitude of reproductive age group women towards cervical cancer and its prevention in Gondar town.

**Methods:**

A descriptive community based cross-sectional study was carried out. An interviewer-administered questionnaire was employed for data collection. A multistage sampling technique was employed to select the study participants. Descriptive statistics like frequency, mean and percentage were computed using SPSS version 20 software program.

**Results:**

Seven hundred and seventy women (*n* = 770) participated with a response rate of 100%. More than half, (65.1%) of the participants claim hearing of cervical cancer. However, majority (> 80%) of them lack knowledge that HPV is a causative agent of cervical cancer which is extremely worrying as the most important way to prevent cervical cancer is blocking HPV infection. Of those who had heard of it, only 107 (21.4%) said they have heard about Pap smear test. From them, less than half, 47 (43.9%) said that an apparently healthy woman should undergo the test at least three times in her life. This means in addition to the lack of information about the test, majority of those who had heard about it didn’t know how many times they should have the test in their life. Overall, only 153 (19.87%) of the participants were found having a good knowledge of cervical cancer and its prevention.

**Conclusion:**

The overall knowledge of women towards cervical cancer was inadequate. On the other hand, those who had heard about it had a somewhat encouraging attitude. Mass media was the major source of information. But, any public health problem cannot be solved in isolation. Hence, initiating large-scale awareness campaigns is recommended.

## Background

Cervical cancer is a cancer of the cervix, the organ connecting the uterus and the vagina. It is predominantly caused by human papilloma virus (HPV) which is a sexually transmittable infection-causing pathogen. Therefore, effective interventions on prevention of HPV infections can prevent cervical cancer [1, 2].

Despite its preventable nature, globally cervical cancer is regarded as the third most common form of cancer among women after breast and colorectal cancer [3–5]. The women of poorer communities are mostly affected by the disease. It is evidenced that, approximately 83% of the world’s new cases and 85% of all cervical cancer deaths reported are from developing countries [1].

The highest incidence rate of cervical cancer was observed in Guinea with nearly 6.5% of women developing cervical cancer before the age of 75 years. It affects women < 45 years more than the other major cancers [5]. It is also the leading cause of cancer deaths in Eastern and Central Africa. Most of these deaths can be prevented through universal access to comprehensive cervical cancer prevention and control programs which can potentially reach all girls with HPV vaccination and all women who are at risk with screening and treatment for pre-cancer [1, 5, 6].

Persistent infection with around 15 high-risk HPV types is the major risk factor for cervical cancer with HPV-16 and HPV-18 infections accounting for about 70% of the total cases. Multiple sexual partners, younger age at first sexual intercourse, early marriage, poor dietary habit, immune suppression, and cigarette smoking also serve as risk factors to the HPV persistent infection and progression to cancer [1, 6, 7].

A study on global cancer transitions according to the human development index reveals that cervical cancer is estimated to be more common than both breast and liver cancer [8]. The study also suggests that rapid socio-economic transition in many countries might reduce infection-related cancers. However, this might be replaced by an increasing number of new cases that are more associated with reproductive, dietary, and hormonal factors [1, 8].

The burden of cervical cancer is reasonably low in the developed countries of the world [9]. However, the situation is quite the reverse in developing countries. While the incidence is decreasing in the former, it is on the increase in the latter [7, 9, 10]. In most parts of Sub-Saharan Africa, South America, the Caribbean, and Southern Asia, cervical cancer is the leading cause of cancer death and premature death among women [7].

Sub-Saharan Africa is the region with the highest incidence of cervical cancer in the world with concomitant high mortality affecting women at their prime. This is a source of great concern considering the fact that cervical cancer is preventable and curable using currently available methods [1, 6]. The onset of HIV/AIDS epidemic that is highest in the region has also raised the problem of cervical cancer to a serious level [6].

According to the 2009 world health organization report, cervical cancer ranks as the second most common cancer among women in Ethiopia [11]. The mean outpatient cost per patient for cervical cancer in Ethiopia is estimated to be $407.2. The mean inpatient cost for hospitalized patients was also estimated to be $404.4. The average direct inpatient cost was $329 and for every additional day of inpatient hospital stay, there was an estimated daily incremental inpatient cost of $4.2. This is very high and unimaginable for many patients to get treatment considering the socioeconomic status of the people [12].

Various studies in different countries show differences in women’s knowledge and attitude regarding cervical cancer and its prevention. Unlike developed nations, in developing countries, women had a poor level of knowledge towards cervical cancer and its prevention [13–15]. A significant direct relationship was also found between women’s knowledge and attitude towards cervical cancer and its prevention, and subsequent utilization of Pap smear test in some studies [16–18].

A very low rate of cervical cancer screening tests is reported across literatures in low and middle-income countries [15, 19–22]*.* A study on health-seeking behavior of patients with cervical cancer in Ethiopia also revealed that women had a very low awareness of cervical cancer and they mostly prefer traditional remedies as a treatment option for the early stages of the disease. According to this study, the barriers to seeking any type of treatment identified were lack of awareness and access to proper health services. It also showed, women with cervical cancer were excluded from society and received poor emotional support [23].

Even though cervical cancer is a growing problem in Ethiopia in terms of morbidity, mortality, cost, and suffering, it has been neglected. To this day there is no national cancer control program and there is no cancer registration process. As a result, there is no morbidity and mortality data available to convince policymakers on this issue [6, 24].

Currently, Ethiopia is trying to expand cervical cancer screening programs and cancer treatment centers in different parts of the country [24]. Despite this effort studies on women’s knowledge and attitude regarding cervical cancer in Ethiopia is limited. Hence, this study aimed to assess reproductive age group women’s level of knowledge and attitude towards cervical cancer and its prevention in Gondar town.

## Methods

### Study area, design and period

A descriptive community based cross-sectional study was carried out in Gondar town. Gondar is the capital city of the North Gondar administrative zone of Amhara region, which is 738 km far from Addis Ababa, the capital city of Ethiopia. According to the 2007 census, Gondar has a total population of 206, 987, of whom 98,085 are males and 108,902 females living in 21 kebeles of the town. The study was conducted at four randomly selected kebeles found in the town from May 1–30, 2014 [25].

### Study population

The study populations were all reproductive age group women who were living in four randomly selected kebeles of Gondar town.

### Eligibility criteria

All reproductive age group women (15–49 years old) who were living in four randomly selected kebeles in Gondar town were included in the study.

### Sample size determination

The sample size for this study was determined by using the single population proportion formula considering the assumptions: The proportion of reproductive age group women having adequate knowledge regarding cervical cancer being 50% (Since there was no previous study in the study area). Level of significance 5% (α = 0.05), Z α/2 = 1.96 and margin of error 5% (d = 0.05). Adding a 10% non- response rate and multiplying by 2 (design effect) the total sample size required for this study appeared to be 770.

### Sampling technique

A multistage sampling technique was employed to select the required samples. First, each of the 21 kebeles found in the town were taken as a cluster. In these clusters, four kebeles were selected by using the lottery method. Then, in these selected kebeles, the number of study participants was assigned proportionally. Finally, a systematic random sampling technique was employed to select study participants from the selected kebeles using house numbers as a unit of selection.

### Variables

The outcome measures of this study were knowledge (which was classified as good and poor knowledge) and attitude (which in turn was also classified as favorable and unfavorable attitude) of reproductive age women towards cervical cancer. The independent variables included socio-demographic characteristics and related issues (age, religion, duration of stay in Gondar town, educational status, belief, occupation, income and exposure to mass media).

### Data collection technique and tools

An adapted and structured, pretested, interviewer-administered questionnaire was employed to collect data from the participants [26]. First, the questionnaire was prepared in English and it was translated into Amharic, the local language, and then back to English to check its consistency. Each correct response received 1 point for knowledge and attitude questions and the scores were transformed into a percentage for interpretation of the results. The mean scores were utilized as a cut of point to describe the women’s level of knowledge and attitude regarding cervical cancer and its prevention. One supervisor and two data collectors, who had BSc degree in nursing, were recruited to assist in the data collection process. The data collectors and the supervisor were trained for one day on how to facilitate the data collection process, prevent errors, keep privacy and confidentiality and other ethical issues.

### Data quality assurance

To ensure the quality of data, the questionnaire was pretested on 5% (39) of the sample size among selected females of reproductive age in Gondar town at kebele 11 from the unselected kebeles in the sampling process. The content and face validity of the questionnaire was done in previous studies [26, 27]. In this study, the Kuder-Richardson 20 (KR-20) reliability coefficient for the knowledge questions was 0.86. On the other hand, the Cronbach’s Alpha reliability coefficient for the attitude questions was 0.92. All the necessary amendments were made on; the instructions, contents, order and grammatical issues. All data were checked for completeness, accuracy, clarity, and consistency by the supervisors and the principal investigator each night after the data were collected. Double data entry and validation were performed and the data were intensively cleaned before analysis.

### Data processing and analysis

The data were coded and entered into a computer using Epi-data 3.1 Statistical program and were exported to SPSS Version 20 for further analysis. Data was processed and cleaned to minimize entry errors, and for outliers and missing values. Then, descriptive statistics like frequency, mean, and percent were computed for the study variables using SPSS version 20 software program. Participant’s knowledge was analyzed and classified as good if a woman scores a result equal to or above the mean score level for questions used to measure knowledge and poor if a woman scores a result below the mean score level for questions used to measure knowledge. Similarly, participants were classified as having a favorable attitude if a woman scores a result equal to or above the mean score level and, those who answer below the mean score were classified as having an unfavorable attitude towards cervical cancer and its prevention. Finally, the result is summarized and presented using texts and tables.

## Results

### Socio demographic characteristics of participants

Seven hundred and seventy women (*n* = 770) participated in the study with a 100% response rate. Overall, 35.3% of women were within the age group of 24–32 years old. Most of the study participants (75.2%) had lived in Gondar town for more than five years. Majority of the study participants (86.8%) were Orthodox followers, followed by 11.7% Muslims. Among the study participants (54.5%) were married. Virtually, 717 (93.1%) participants were from the Amhara ethnic group. Of the participants, 82 (10.6%) claim they can’t read and write. The major proportion, 264 (34.3%) had a monthly income of < 800 Ethiopian Birr (Table 1).

**Table 1:** Socio demographic characteristics of participants in Gondar town, Ethiopia, 2014 (*n* = 770)

Variables	Response	Frequency	Percentage
Age	15–23	255	33.1
	24–32	272	35.3
	33–41	149	19.4
	42–49	94	12.2
Length of stay in the town	≤5 years	191	24.8
	> 5 years	579	75.2
Religion	Orthodox	668	86.8
	Muslim	90	11.7
	Protestant	6	0.8
	Catholic	1	0.1
	Others	5	0.6
Marital status	Married	420	54.5
	Single	253	32.9
	Divorced	64	8.3
	Widowed	33	4.3
Ethnicity	Amhara	717	93.1
	Tigray	40	5.2
	Oromo	11	1.4
	Others	2	0.3
Educational level	Can’t read and write	82	10.6
	Read and write	67	8.7
	Primary	126	16.4
	Secondary	184	23.9
	Preparatory	61	7.9
	Diploma or degree	250	32.5
Occupation	House wife	196	25.5
	Student	154	20.0
	Commercial sex worker	7	0.9
	Governmental employee	187	24.3
	Merchant	149	19.4
	Others	77	10.0
Monthly income	≤ 800 ETB	264	34.3
	800–1200 ETB	169	21.9
	1200–1600 ETB	163	21.2
	≥ 1600 ETB	174	22.6

Key: ETB - Ethiopian birr

### Participants knowledge regarding cervical Cancer and its prevention

In this study, it is found that more than half, 501 (65.1%) of the participants had heard about cervical cancer. Of those who had heard about cervical cancer, the largest number, 206 (41.1%) had heard from mass media. The mean knowledge score was 3.21 (Standard deviation = 3.88) and the median was 2. This study reveals that only 153 (19.87%) of the participants had good knowledge regarding cervical cancer and its prevention.

The participants were asked whether they knew the risk factors that can lead to cervical cancer and from those who have heard about it (501), 299 (59.7%) of them replied they didn’t know the risk factors. Risk factors such as sexually transmitted disease, smoking, sex with multiple partners’, family history of cervical cancer and others such as giving frequent births were asked. Majority, 408 (81.4%) of the participants didn’t know whether cervical cancer is caused by HPV or not.

Similarly, 401 (80%) of the participants who had heard about it claim they didn’t know the symptoms of cervical cancer. The common symptoms asked were: intra or post-coital bleeding, bleeding after menopause, persistent blood-stained vaginal discharge and lower abdominal pain. Concerning prevention and treatment, only 327(65.3%) and 214 (42.7%) said that cervical cancer is preventable and curable respectively. Regarding screening tests, of the 501 participants who had heard about cervical cancer, only 107 (21.4%) of them said they had heard about the Pap smear test. Of those who had heard about the Pap smear test, less than half, i.e. 47 (43.9%) said that an apparently healthy woman should undergo a Pap smear test at least three times in her life (Table 2).

**Table 2:** Participants knowledge regarding cervical cancer in Gondar town, North West Ethiopia, 2014 (*n* = 770 for the first question and, 501 for the rest)

Variables	Response	Frequency	Percentage
Heard about cervical cancer	Yes	501	65.1
	No	269	34.9
Know the risk factors for cervical cancer	Yes	202	40.3
	No	299	59.7
Knowledge on risk factors of cervical cancer	Sexually transmitted disease	153	75.7
	Smoking	120	59.4
	Having multiple Sexual Partners	135	66.8
	Poor dietary habit	40	19.8
	Early marriage	129	63.8
	Family history of cervical cancer	19	9.4
	Others	18	8.9
HPV is a causative agent of cervical cancer	Yes	93	18.6
	No	408	81.4
Know common symptoms of cervical cancer	Yes	100	20.0
	No	401	80.0
Knowledge on the symptoms of cervical cancer	Intra or Post coital bleeding	58	58.0
	Bleeding after menopause	38	38.0
	Persistent blood stained vaginal discharge	73	73.0
	Lower abdominal pain	33	33.0
	Others	1	1.0
Cervical cancer is preventable	Yes	327	65.3
	No	174	34.7
Cervical cancer is curable	Yes	214	42.7
	No	287	57.3
Heard about pap smear test	Yes	107	21.4
	No	394	78.6
If yes where did you hear about Pap smear for the first time?	Relatives	64	12.8
	Friends	88	17.6
	Health workers	123	24.6
	Mass media	206	41.1
	Others	20	4.0
How many times should a healthy woman undergo pap smear test?	Only once	26	24.3
	Two times only	34	31.8
	At least three times and above	47	43.9

### Participants attitude towards cervical Cancer and its prevention

In this study, it is found that the mean attitude score was 3.86. From the study participants who had heard about cervical cancer, 370 (73.9%) replied, they believe that having multiple sexual partners is a risk factor for cervical cancer. More than half, 318 (63.5%) believed that HIV positivity can increase the chance of getting cervical cancer. On the other hand, 433 (86.4%) didn’t believe that the use of oral contraceptive pills is a risk factor for cervical cancer.

Regarding smoking and early marriage, of those who had heard about it, 338 (67.5%) and 366 (73.1%) believed these conditions are a risk factor for cervical cancer respectively. In line with this, 442 (88.2%) believed that cervical cancer is a major health problem for reproductive-age women and, 253 (50.5%) believed that cervical cancer cannot be detected by early screening before symptoms appear. However, 457 (91.2%) believed that, early detection of cervical cancer is good for treatment outcomes.

Similarly, 391(78%) of those who had heard about it believed that cervical cancer is preventable. Regarding the prognosis, 264 (52.7%) believed that cervical cancer is not curable and the remaining 237(47.3%) believed it is curable. The study also reveals overall more than half, 448 (58.2%) of the study participants had a favorable attitude towards cervical cancer and its prevention (Table 3).

**Table 3:** Participants attitude on test items towards cervical cancer in Gondar town, Ethiopia, 2014 (*n* = 501)

Variables	Response	Frequency	Percentage
Believe having multiple sexual partners is risk factor for cervical cancer	Yes	370	73.9
	No	131	26.1
Believe cervical cancer is transmittable through sexual inter course	Yes	112	22.4
	No	389	77.6
Believe HIV positivity increases the chance of getting cervical cancer	Yes	318	63.5
	No	183	36.5
Believe use of oral contraceptive pill is a risk factor for cervical cancer	Yes	68	13.6
	No	433	86.4
Think that smoking is a risk factor for cervical cancer	Yes	338	67.5
	No	163	32.5
Think early marriage is a risk factor for cervical cancer	Yes	366	73.1
	No	135	26.9
Think cervical cancer is a major health problem for female of reproductive age group	Yes	442	88.2
	No	59	11.8
Think it is possible to detect cervical cancer with early screening before symptoms appear	Yes	248	49.5
	No	253	50.5
Think early detection of cervical cancer is good for treatment out come	Yes	457	91.2
	No	44	8.8
Believe cervical cancer is preventable	Yes	391	78.0
	No	110	22.0
Think it is possible to cure cervical cancer	Yes	237	47.3
	No	264	52.7

## Discussion

This study reveals that more than half, 501 (65.1%) of the participants had heard about cervical cancer. This finding is consistent with a study in Qatar, Niger, and North Korea in which over 85, 72, and 62% claiming being aware of cervical cancer respectively. This reflects in terms of information there is encouraging evidence in these countries and Ethiopia. But, this information is not enough in creating knowledge which is reflected in the three studies and the present study [27–29]. In contrast, the result is higher than a study conducted in South Africa and another study in Ghana which shows that only 42.9 and 30.6% of the participants had heard of about cervical cancer respectively [19, 26].

Mass media was the major source of information (41.1%), which is in line and far more than the South African study in which only 19% hearing about it from the media [26]. This difference might be due to differences in the study population in which the South African study included only university students who are mostly young aged and are not the main risk groups for cervical cancer and possibly not target groups for health education programs regarding it. However, the finding is in contrast with North Korea’s study in which health care providers were the main source of information (69%). This difference might be due to differences in media coverage between the countries, health service access and utilization levels. It might be also due to differences in the study population in which the North Korea study had included both urban and rural women’s [29].

In this study, only 100 (19.96%) of the participants who had heard about it claim they knew the symptoms of cervical cancer. This finding is in line with the North Korea’s study in which less than 40% were aware of the symptoms of cervical cancer. This consistency might be due to the fact that, the overall level of knowledge between the two studies was inadequate [29]. It might be also due to the lack of health education programs regarding cervical cancer which is believed to be the problem of most developing countries [30].

Regarding the risk factors, of those who had heard about it only 153 (30.54%) of the participants claim that sexually transmitted disease is a major risk factor for cervical cancer. This is in line with the Qatar study in which only 24.8% having the same claim [27]. It is also in line with North Korea’s finding which has found that only a very small proportion (3%) knew that cervical cancer is predominantly the result of a sexually transmitted infection [29]. This consistency might again emerge from poor health care access and utilization levels and the lack of large-scale health education programs.

Similarly, only 93 (18.6%) of the participants who had heard about it knew that HPV is a cause of cervical cancer which is very low compared to the South African and a study in Iraq in which around 48.5 and 36.9% of the respondents saying they knew that HPV causes cervical cancer respectively [26, 31]. 174 (34.7%) said cervical cancer cannot be prevented which is much higher compared to the South African study in which only 1.2% of participants claiming cervical cancer cannot be prevented [26]. Both findings show how the knowledge of women in Gondar town is insufficient. These differences might be due to differences in access to health care services, utilization, and health education programs among the countries. It may be also due to differences in the educational level of the study participants.

Participants were also asked to answer whether cervical cancer is preventable and curable. From those who had heard about it, 327 (65.3%) and 214 (42.7%) said that cervical cancer is preventable and curable respectively. This means from all participants, only 327 (42.5%) and 214 (27.8%) knew that cervical cancer is preventable and curable respectively. This is in line with the North Korea’s study which revealed that majority of respondents (64%) did not know that cervical cancer can be prevented [29]. This consistency might again emerge from the inadequate knowledge of participants between the two studies. It might also emerge from the lack of information about the prevention and treatment of cervical cancer. This reflects the hypothesis “the fewer people have the information about a certain problem the lower their knowledge”.

Regarding screening tests, of the 501 participants who had heard about cervical cancer, only 107 (21.4%) of them said they had heard about the Pap smear test. This finding is consistent with the Ghanaian study and with a study in Iraq in which only 3.3 and 28.79% claiming having heard of the Pap smear test and knowing that Pap smear is a test used to detect abnormal cervical cells in the respective studies [19, 31]. However, it is very low compared to the Qatar study which revealed that 76% of women have heard about the Pap smear test. This discrepancy might be due to the study setting in which the Qatar study was institution-based and those who visit institutions are more likely to have information about the test [27]. It might be also due to differences in access and utilization of health care services in the two countries.

Of those who had heard about the pap smear test, only 47 (43.9%) said that an apparently healthy woman should undergo a pap smear test at least three times in her life. This reveals that in addition to the lack of information about the Pap smear test, majority of those who had even heard about it didn’t know how many times and/or how often they should have a Pap smear test in their life. This is in line with the Niger study and a study in Iran which shows that only 50.6, and 44.3% being aware of cervical cancer screening tests respectively [28, 29].

The study had again revealed that overall only 153 (19.87%) of the participants had good knowledge regarding cervical cancer and its prevention. This reflects even majority of those who had heard about cervical cancer didn’t have sufficient knowledge regarding the disease. This is consistent with a study conducted on sexual health status and cervical cancer prevention among 250 Thai couples in which majority, (65.6%) having a low level of knowledge about cervical cancer and its prevention [32]. This consistency might emerge from a lack of information about it. This might be also due to the lack of awareness creation programs regarding cervical cancer. Because chronic diseases like cancer are neglected and the major focus of government in most developing countries including Ethiopia is on infectious disease prevention [30]. It is also consistent with other studies across literatures in low and middle-income countries [14, 31, 33].

This study has also tried to assess the women’s attitude, and of those (501) who had heard about cervical cancer, 370 (73.9%) and 366 (73.1%) believed that having multiple sexual partners and early marriage are risk factors for cervical cancer respectively. This may be attributed to the mere community belief that any unsafe sexual behavior is a risk for diseases. Because the community believes that people having multiple sexual partners always get punishment for their sins from God. Similarly, 442 (88.2%) believed that cervical cancer is a major health problem for reproductive age group women. This may again be the result of a mere belief that any cancer is a serious health problem.

In line with the above finding, 248 (49.5%) believed that cervical cancer can be detected by early screening before symptoms appear which is lower than the Qatar study which conducted among 500 women visiting primary health care, in which 312 (62.4%) believed that cervical cancer can be detected with Pap smear test before symptoms appear. This difference might be due to the fact that, the Qatar study was institution-based and those who visit health institutions are more likely to get information about the Pap smear test [27].

Of the 501 who have heard about cervical cancer, 457 (91.2%) believed that early detection of the disease is good for treatment outcomes. This is higher than the Qatar study in which, 64.8% having the same belief [27]. This is quite surprising. But, it might be due to the community’s perception that if a disease is once recognized by physicians, then the outcome is usually good. This in turn might be the result of inadequate knowledge of the community about the medical world [34].

Regarding the prevention and treatment outcome, 391(78%) believed that cervical cancer is preventable which is higher than a descriptive cross-sectional study conducted on the knowledge and attitude about cervical cancer in different countries at different levels of development in which 62% believed it was possible to prevent cervical cancer [35]. Less than half, 237 (47.3%) believed that cervical is curable which is very much lower than the study in Qatar in which 412 (82.4%) believed that cervical cancer is curable. This discrepancy could be due to the fact that in this study the knowledge of women about cervical cancer and its prevention was very low compared to the Qatar study [27].

This finding is also higher and in controversy with the finding in the knowledge section in which only 327 (65.3%) and 214 (42.7%) saying yes and the remaining no/don’t know for the questions “is cervical cancer preventable and curable” respectively. This might emerge from those participants who said they don’t know in the knowledge section for the questions but having a positive belief in the attitude section. Because, no and don’t know were merged into “no” during the analysis phase.

The study also shows that more than half, 448 (58.2%) of the study participants had a favorable attitude which is higher than the Qatar study which revealed that majority, 63.2% having an unfavorable attitude regarding prevention and screening test of cervical cancer respectively [27] *.* This finding together with the other findings indicates, despite the low level of women’s knowledge regarding cervical cancer and its prevention, their attitude is somewhat encouraging. It is also in line with other studies in Nepal and Iraq which reported that women had a positive attitude towards cervical cancer and Pap smear test [14, 33, 36]. But, the alarm here is that the result might emerge from mere optimism of study participants.

### Limitation

This study was limited to Gondar town only due to constraints of time and funds. It would have been better if the rural women were included and differences in knowledge and attitude regarding cervical cancer were assessed among the two groups.

## Conclusion

The overall knowledge of participants towards cervical cancer and its prevention was inadequate. The majority, > 80% of the participants lack knowledge that HPV is a causative agent of cervical cancer. This is extremely worrying as the most important way to prevent cervical cancer is blocking HPV infection. Furthermore, only 21.4% have heard about cervical cancer screening tests. Of them, less than half, 47 (43.9%) said that an apparently healthy woman should undergo the test at least three times in her life. On the other hand, those who have heard about cervical cancer had a somewhat encouraging attitude.

Mass media was the major source of information regarding cervical cancer. However, any public health problem cannot be solved in isolation. Thus, governmental and non-governmental organizations and other concerned bodies need to work in collaboration to increase the level of women’s general awareness of cervical cancer and its prevention. Besides, the government should work on collaborating health institutions with other sectors on awareness creation campaigns.

## Data Availability

Data supporting the conclusions of this article will be available on reasonable request to Ayelign Mengesha.

## Abbreviations

BSc: Bachelor of Science
ETB: Ethiopian birr
HIV: Human immunodeficiency virus
HPV: Human papilloma virus
Pap smear: Papanicolaou smear test
SPSS: Statistical package for social sciences
